# Multi-Species Genomics-Enabled Selection for Improving Agroecosystems Across Space and Time

**DOI:** 10.3389/fpls.2021.665349

**Published:** 2021-06-23

**Authors:** Marnin D. Wolfe, Jean-Luc Jannink, Michael B. Kantar, Nicholas Santantonio

**Affiliations:** ^1^Plant Breeding and Genetics Section, School of Integrative Plant Science, Cornell University, Ithaca, NY, United States; ^2^United States Department of Agriculture – Agriculture Research Service, Ithaca, NY, United States; ^3^Department of Tropical Plant and Soil Science, University of Hawai‘i at Mānoa, Honolulu, HI, United States; ^4^School of Plant and Environmental Sciences, Virginia Tech, Blacksburg, VA, United States

**Keywords:** genomic selection, agroecosystems, intercropping, polyculture, breeding, ecosystem services

## Abstract

Plant breeding has been central to global increases in crop yields. Breeding deserves praise for helping to establish better food security, but also shares the responsibility of unintended consequences. Much work has been done describing alternative agricultural systems that seek to alleviate these externalities, however, breeding methods and breeding programs have largely not focused on these systems. Here we explore breeding and selection strategies that better align with these more diverse spatial and temporal agricultural systems.

## Introduction

Climate change and human population growth are continually increasing demand for food and services from agroecosystems. To meet these demands sustainably, food production must be intensified. These challenges require innovation and diversification in agroecological-systems design and management (Runck et al., 2014; Litrico and Violle, 2015; Henkhaus et al., 2020). Today the dominant form of agriculture across the globe consists of large acreages of monoculture production (Crews et al., 2018). Monocultures provide uniformity in plant architecture and maturation, facilitating efficient mechanical harvesting and minimizing human labor.

The combination of new crop types, synthetic fertilizers, and irrigation has dramatically increased crop production per unit area while simultaneously sparing land for natural ecosystems (Burney et al., 2010). This has come at an environmental cost. Increases in water and nutrient pollution, vast new energy and fossil fuel requirements to produce fertilizers, and steady losses of crop diversity. Maintaining or intensifying production while decreasing external inputs and soil disturbance (i.e., tillage) requires cropping systems that are more spatially (intercrops, polycultures) and temporally (rotations, relays) diverse, and in many cases include longer-lived (i.e., perennial) species (Lovell and Taylor, 2013).

Modern plant and animal breeding is a predictive, data-driven, multi-disciplinary science. Statistical prediction methods that leverage genomic and phenomic data (e.g., drone-based hyperspectral imaging) are greatly accelerating the rate of population genetic improvement (Jannink et al., 2010; Hickey et al., 2017; Voss-Fels et al., 2019; Krause et al., 2020). Decision support tools based on these technologies are now available to large-acreage monoculture systems. Transitions to new agricultural practices are expensive and require agronomy and operations research. Nevertheless, state-of-the-art breeding is largely focused on individual species and the development of single genotypes, for their single-season monoculture performance.

Indeed, breeding and agronomy typically operate on vastly different scales of genetic variation. Breeders evaluate hundreds or thousands of genotypes in only limited combinations of management, environmental and cropping system variations. Agronomists and agroecologists, in contrast, test diverse cropping and management practices, but against relatively few, “representative,” cultivars of each species.

A sustainable future for food is a *highly* multi-objective optimization problem. At the landscape level there is incredible heterogeneity, comparable in magnitude to variability in yearly climate patterns. Therefore creating sustainable landscapes that serve multiple functions requires combining food and non-food crops as well direct and *indirect* services from landscapes. Diversified agroecosystems are expected to exhibit better sustained productivity and multifunctionality over long time periods, borne out in theory from economics (Goerner et al., 2009; Paut et al., 2020), ecology (Holling, 1973), and agriculture (Schipanski et al., 2016). The productivity-diversity relationship is expected to depend on the degree of resource-use niche complementarity vs. redundancy and the nature of interspecific interactions (Brooker et al., 2015; Bowles et al., 2020; Tamburini et al., 2020). However, these robust results have yet to be widely adopted in the breeding industry and when they are, they rarely use state-of-the-art tools. Despite strong evidence for the benefits of cropping-system diversification (Tamburini et al., 2020) and calls in the literature (Brooker et al., 2015; Litrico and Violle, 2015; Sampoux et al., 2020), the improvement of complex multi-species, multi-genotype systems has not been a priority.

Instead of breeding to improve monoculture yield of single crops in isolation, we propose optimizing multiple interacting species and genotypes. We seek to enable joint-selection to improve the performance of the cropping system across time and space. We argue that the largely disparate literature on diversification and agroecological intensification, genomics and phenomics-enabled selection collectively indicate the advantage of developing prediction and selection strategies to tackle the multiple outputs of cropping systems and their responses to environmental changes. This represents an important frontier in agriculture and strategies need to be devised for maintaining and enhancing beneficial interactions while reducing or avoiding negative ones.

## Joint-Search of Multiple Gene Pools for Adaptive Interspecific Interactions With Genomic Prediction

Investigating all possible combinations of genotypes between any diverse set of germplasm from one species (or population), and a diverse set of another interacting species (or population), is intractable. Borrowing methodology from maize hybrid breeding [reciprocal recurrent selection (Comstock et al., 1949)], (Wright, 1985) developed an interspecies selection scheme, which partitions plot-level performance into main effects for each species (general mixing ability; GMA) and an interaction (specific mixing ability; SMA) (Federer, 1993; Forst et al., 2019; Sampoux et al., 2020; Haug et al., 2021). We note that a GMA is estimated for each genotype of each single crop, but that these GMAs refer to emergent plot-level properties (e.g., erosion protection) that can only be measured on crop combinations. The intractably large genotype-by-genotype *inter*specific interaction landscape can be enumerated and the “best” *inter*specific genotypic combinations can be identified using numerical optimization and genomic prediction. Rather than attempting to test all possible combinations, accessions-to-be-phenotyped should be algorithmically chosen, similar to modern approaches in hybrid breeding (Zhao et al., 2015) such that genetic variation in each species is tested against a representative sampling of variation in the other species.

The application of genomic prediction to unobserved intercrop combinations has recently been suggested (Annicchiarico et al., 2019; Bančič et al., 2020). Genomic prediction has not been applied using these models. Empirical estimates of GMA/SMA are scarce and have only occasionally detected statistically significant SMA (Collins and Rhodes, 1989; Knott and Mundt, 1990; Federer, 1993; Holland and Brummer, 1999; Lopez and Mundt, 2000; Forst et al., 2019; Haug et al., 2021). Approaches to date have been constrained to individual species productivity in the immediate environment of the other species rather than accounting for total agroecosystem productivity through time.

**FIGURE 1:**
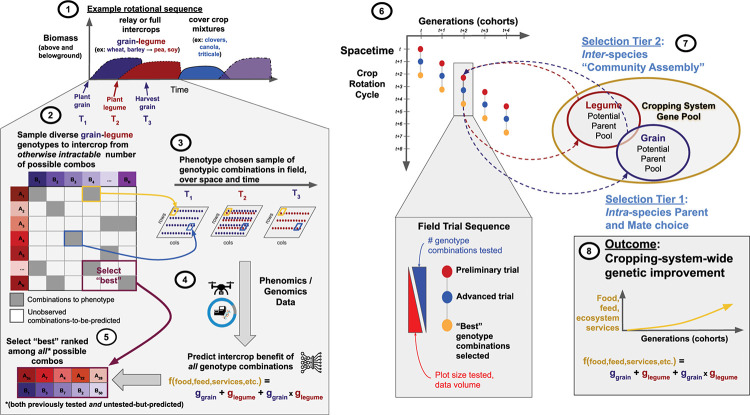
Rapidly exploring the adaptive landscape of *interspecific* genomic-interactions to find combinations that optimize system-wide benefit. (1) An example vegetation sequence. (2) Zoom in on the grain-legume portion. The grid of tiles represents all possible combinations of grain-genotype-by-legume-genotype among representative (“training”) populations for each species, all genotyped genome-wide. Diverse combinations are sampled (gray tiles); each genotype/species is chosen at least once. Blue/yellow tiles and arrows illustrate how chosen grain-legume intercrops are spatiotemporally combined in the field. (3) Three timepoints (T_1_, T_2_, T_3_) in sequence. At T_1_ grain is planted, followed by relay intercropping (interplanting) the legume at T_2_. At T_3_ the grain is harvested and the legume is left to mature. The sequence continues depending on the system. (4) Phenomics data are collected over time at plot-resolution. Prediction of the performance [f(food, feed, services, etc.)] of grain (g_*grain*_), legume (g_*legume*_) and their spatiotemporal combination (g_*grain*_ × g_*legume*_) is used to enable selection (5) of the “best” among *all* combinations, both previously tested [gray tiles] *and* untested-but-predicted [white tiles]. (6) Iterative (breeding) scheme. Steps 1–4 take place *within* each dot: “Preliminary trial” (red dot-steps 2–4), followed by “Advanced trial” (blue dot-step 5+), terminating in the identification of new “Best” intercrop combinations (orange dot). (7) The cropping system gene pool comprises all relevant germplasm of e.g., grain+legume. Dashed arrows represent recurrent selection: Tier 1 = *intra-*specific selection of genotypes as parents to cross; Tier 2 = *inter-*specific selection of genotypes to intercrop/field test, which takes place at entry to “Preliminary” *and* “Advanced” trials. (8) Over time and across successive cohorts of tested intercrops system-wide improvement is achieved.

Genomic and phenomic prediction poses an exciting opportunity to develop what we describe below as a multi-tiered selection scheme. Figure 1 shows an example of how this can be operationalized, using a no-till grain-legume sequence example and an experimental design that develops a profile of phenomic and genomic variation within- and among-species across space and time. The iterative field evaluation procedure has the potential to enable directed co-improvement of all species and their interaction for overall system performance.

## Multi-Tiered Selection: Genes to Cropping Systems

Consider that the phenotype of any individual is the response to an environment that is shaped by the other organisms present in that environment, both current and past. This highlights that, from the perspective of prediction, genome-by-genome interactions (G × G) are a special case of the genotype-by-environment (G × E) interaction where the covariance of the environment *is* the interacting species’ genotypic covariance. Typically, phenotypic evaluation is done at particular locations under targeted management conditions in an effort to “control” the environment under which focal species are observed and for which they are selected. In the general case, the objective function, *f*[*G*_*ij*_ | *E*(*G*_*i*__’__*j*__’_, *t*, *s*)], assigns a genetic value to *j*th individual, of the *i*th species (*G*_*ij*_) conditional on the environment, *E*, which is itself a function of other species (*G*_*i*__’__*j*__’_) in the system, space (*s*) and time (*t*). In the classic case, *G*_*i*__’__*j*__’_, *t* and *s* are all held constant or partitioned to the error term and single season yield is the objective function to be optimized. When the other species in the system, space and time are simultaneously taken into account, we develop a generalized agroecosystem selection scheme.

Prediction and selection strategies that leverage genomic/phenomic tools to address more than single-species, single-season, monoculture evaluation should be a major frontier for future research and development. We highlight that there are multiple levels or “tiers” of selection, which when considered jointly enact agroecosystem improvement. Importantly before selection begins, the goals must be defined (Table 1). The objective at Tier 1 is *intra*specific population improvement, which is addressed simultaneously across each species to effect co-adaptation of the germplasm pools. Tier 1 evaluation identifies promising parents and matings. At Tier 2, selection is focused on predictions of performance of the combination over space and time (e.g., of the intercrop overall). The objective at Tier 2 is to select the “best” *inter-specific (or intra-*specific) genotype combinations to assemble in space over time, i.e., to release to farmers that maximize farm profit and ecosystem function.

**TABLE 1:** Potential cropping system applications, their associated interactions and agroecosystem objectives.

Cropping system/Agroecosystem	Interactions	Objectives
**Temporal rotations** E.g., following corn with soy.	**Indirect. Legacy effects.** Current (past) crops condition (esp. soil) environment for future crops.	**Minimize or Reduce:** loss of soil N, non-prod. time, non-target/weed species, runoff, nutrient input, herbicide pesticide
**Relay intercrops** E.g., soy planted between rows of maturing barley without tillage.	**Direct and Indirect effects.** Maturing crops influence microenvironment (weed suppression, shade, soil moisture, architectural support) to young crops *plus* legacy effects on subsequent crops.
**Full intercrops** Planted together. Harvested separately, or separated at harvest. E.g., three-sisters (corn, beans, squash), maize-peanut, guava/mango/cowpea, banana and root crops (sweet potato, yam, cassava), sugarcane-sweet potato, orchard and agroforestry alleys		**Maximize or Increase:** retention of soil C, net primary productivity, germination
		↑↓		
**Species mixtures/polycultures** Harvested together. E.g., mixtures of grasses, legumes and mustards used as cover crop mixtures/green manures, biofuel/biomass crops, perennial plantings to reduce erosion/runoff		**Max avg. profit,** **Min var. Profit**

Determining the selection goals for Tiers 1 (breeding decisions) and 2 (intercropping decisions) are the landscape-scale, cropping-system wide properties, considered over multiple seasons, species and performance indicators, which are community- and market-defined. Thus, while Tier 1 can be viewed as effecting co-adaptation of crops to an overall diversified cropping sequence, Tier 2 includes optimization of and potential specific decisions about the sequence of cropping in space and time. This framework can thus be adapted to both generally and specifically diversified spatiotemporal configurations (e.g., cropping sequences, planting densities) for any potential product profile of the agroecosystem that is to be considered.

## Broader Implications for Changing the Landscape

There are many important potential applications, which this framework can address. Each of these represent different multi-objective optimization problems with respect to competition and interactions, which need to be defined and have been, in some cases, reviewed elsewhere (Picasso et al., 2008; Brooker et al., 2015; Kantar et al., 2016; Ryan et al., 2018; Duchene et al., 2019). Table 1 provides brief examples of applications, the types of interactions to improve and potential benefits.

Theory and agronomic knowledge are available to help understand how different crop species should interact, but optimal multi-species selection strategies have not been developed. Selection and optimization strategies need to balance positive effects against potentially negative ones including financial, human health and environmental costs of managing such systems. While farmers already practice crop rotation, they do not have access to varieties explicitly adapted to one another beyond their ability to meet the basic phenology and management requirements. Identification of the cropping systems and selection indices that support stated multi-species system-level goals is critical and will need careful consideration. We suggest that involvement of farmers and other stakeholders through participatory breeding approaches will be an important component for success (Runck et al., 2014; Lammerts van Bueren et al., 2018). Stakeholder and policy support throughout the process is essential to ensure resources and acreage are not overspent and that cropping system selection indices are constructed in such a way that the agricultural products that are developed perform verifiable services that are collectively desirable.

The framework described here aims to facilitate the design, development and marketing of co-cultivars. These seed “packages” would consist of combinations of varieties selected to optimize the agroecosystem over the long-term, for objectives beyond single-season, single-crop yield.

## Data Availability Statement

The original contributions presented in the study are included in the article, further inquiries can be directed to the corresponding author/s.

## Author Contributions

MW led the writing and developed the figure and table. MK contributed substantially to writing. MW, J-LJ, MK, and NS contributed to the conceptual development, read, commented, and approved the manuscript for submission. NS supervised the development of the article. All authors contributed to the article and approved the submitted version.

## Conflict of Interest

The authors declare that the research was conducted in the absence of any commercial or financial relationships that could be construed as a potential conflict of interest.
